# Stakeholder Perspectives on Health System Readiness for Integrating Artificial Intelligence into Public Health in Saudi Arabia

**DOI:** 10.3390/healthcare14183008

**Published:** 2026-09-14

**Authors:** Sultan Alsahli

**Affiliations:** Department of Health Administration and Hospitals, College of Public Health and Health Informatics, Umm Al-Qura University, Makkah 24382, Saudi Arabia; sssahli@uqu.edu.sa

**Keywords:** artificial intelligence, public health, health system readiness, digital health, data governance

## Abstract

**Background/Objectives**: Although artificial intelligence (AI) has the potential to strengthen disease surveillance, predictive analytics, preventive interventions, and population-level decision-making, its successful integration requires health systems to be adequately prepared across technical, workforce, governance, and organizational dimensions. Saudi Arabia’s rapid digital health transformation provides a unique national context for exploring these readiness conditions. This study explored stakeholder perceptions of conditions relevant to health system readiness for integrating AI into public health in Saudi Arabia, including perceived challenges, implementation requirements, and strategic opportunities. **Methods**: A qualitative descriptive study was conducted using semi-structured interviews with 32 participants, including healthcare professionals, health informatics experts, and policymakers or healthcare planning stakeholders in Saudi Arabia. Participants were purposively selected based on their relevant professional experience. Data were analyzed using Braun and Clarke’s six-phase reflexive thematic analysis. **Results**: Four major themes were identified: (1) infrastructure readiness, reflecting progress in digital health platforms alongside persistent interoperability and data-integration challenges; (2) workforce capacity, highlighting limited AI literacy and the need for practical, role-specific training; (3) data governance and ethics, emphasizing privacy, accountability, algorithmic bias, and the need for greater regulatory clarity; and (4) perceived strategic opportunities, including preventive care, population health monitoring, disease surveillance, and decision support. The first three themes reflected conditions perceived as relevant to health system readiness, whereas the fourth captured anticipated applications and potential benefits rather than a dimension of current readiness. Overall, participants perceived substantial national strategic commitment and digital health development while identifying important implementation challenges related to interoperability, workforce capability, governance, and regulation. **Conclusions**: Participants’ accounts suggest that responsible AI integration into public health requires coordinated attention to interoperable infrastructure, workforce competencies, governance mechanisms, regulatory oversight, and human-centered implementation. A phased implementation approach, supported by role-specific AI training and clear governance and regulatory frameworks, may help translate national AI ambitions into safe and effective public health practice. These findings may inform policy development, workforce planning, and implementation strategies for responsible AI integration in Saudi Arabia and other regions whose health systems are undergoing rapid digital transformation.

## 1. Introduction

Artificial intelligence (AI) is increasingly shaping the future of public health by enabling more advanced approaches to surveillance, prediction, decision support, and population health management [1,2]. In contrast to traditional digital health systems that mainly support documentation, communication, and service delivery, AI-based systems can process large and complex datasets to identify patterns, generate predictions, and support timely decision-making [1]. These capabilities are particularly relevant to public health, where effective action depends on the rapid interpretation of data from multiple sources, including electronic health records, laboratory systems, public health registries, clinical information systems, and community-level data. AI therefore has the potential to strengthen core public health functions, including disease surveillance, outbreak detection, risk stratification, emergency preparedness, preventive care, and resource allocation [1,2].

The COVID-19 pandemic further highlighted the importance of digitally enabled public health systems [1]. During the pandemic, health systems worldwide faced major challenges related to data fragmentation, delayed reporting, limited predictive capacity, and rapidly changing service demands [3,4]. At the same time, the pandemic demonstrated the value of digital technologies and data-driven tools in supporting epidemic modeling, contact tracing, risk assessment, and public health planning [5]. In Saudi Arabia, this digital response included tools such as the Tawakkalna and Tabaud applications, which supported functions such as health-status reporting, movement management, contact notification, and COVID-19 surveillance [6,7]. These initiatives demonstrated the practical value of integrated digital platforms for supporting population-level public health monitoring and response during a national health emergency. While this experience increased global interest in AI as a complementary tool for strengthening public health preparedness and response, the transition from AI innovation to sustainable, system-wide implementation remains complex. The value of AI depends not only on the availability of algorithms but also on the readiness of health systems to adopt, govern, and integrate these technologies safely and effectively [8].

Health system readiness for AI in public health is a multidimensional concept that extends beyond general digitalization. Digital transformation refers broadly to the adoption and integration of digital technologies, platforms, and data-enabled services, whereas AI readiness concerns whether a health system has the specific technical, human, governance, ethical, and regulatory capacities required to implement AI safely, effectively, and sustainably. In the context of public health, key dimensions of AI readiness therefore include digital infrastructure and interoperability, data quality and governance, workforce capability, regulation and ethics, and organizational capacity for implementation. International guidance emphasizes that AI should be implemented through strong data foundations, trustworthy governance, stakeholder engagement, and safeguards that protect autonomy, safety, transparency, accountability, equity, and human rights [9,10]. These requirements are particularly important in public health because AI may inform decisions affecting large populations and vulnerable groups. Readiness therefore depends on secure, representative, and interoperable data; clear frameworks for data sharing, privacy, and accountability; and a workforce capable of interpreting AI outputs, recognizing limitations and risks, and integrating AI insights with professional judgment [1,2]. Establishing these foundations is essential to ensure that AI strengthens, rather than replaces, human decision-making.

In this study, readiness was therefore treated as a multidimensional sensitizing concept rather than as a formally measured index or readiness score. Because the study aimed to explore stakeholder perceptions rather than validate or apply a formal readiness assessment instrument, no single readiness framework was adopted; instead, the identified dimensions were used as sensitizing domains to guide data collection and interpretation. The analysis focused on the dimensions addressed in the interviews, particularly infrastructure and interoperability, workforce capability, data governance and ethics, regulatory considerations, organizational capacity, and implementation conditions. The study does not claim to provide an objective assessment of all possible readiness domains, including financing or leadership capacity, as these were not systematically examined as distinct domains.

Saudi Arabia provides an important context for examining AI readiness in public health given its strong national commitment to health system transformation, digital innovation, prevention, and improved quality of care under Vision 2030 [11,12,13]. The Health Sector Transformation Program has emphasized the development of a more integrated and effective health system, supported by e-health services and digital solutions, while the Saudi Data and Artificial Intelligence Authority has advanced the national data and AI agenda through the National Strategy for Data and AI [13]. Several national initiatives have illustrated this transformation in practice. Seha Virtual Hospital has expanded virtual specialist care and incorporated AI-enabled applications, while the Nafis unified health file supports health data exchange across healthcare providers. In addition, SDAIA and the Ministry of Health have established a Center of Excellence for AI in Health to strengthen health data standards, AI capabilities, and the development of healthcare AI solutions [14,15]. These developments have created a supportive policy environment for AI adoption, reinforced by progress in electronic health systems, virtual care, health information exchange, and national digital platforms. However, these achievements primarily reflect progress in digital transformation and should not be interpreted as evidence of comprehensive AI readiness. Digital transformation provides important technological and data foundations, whereas AI readiness additionally requires interoperable and high-quality data, workforce capability, appropriate governance, regulatory clarity, ethical safeguards, and organizational capacity to integrate AI into practice. This distinction is consistent with recent analysis of AI in clinical medicine, which highlights substantial opportunities for improving diagnostic processes, therapeutic decision-making, and patient safety, while also emphasizing challenges related to data privacy, algorithmic bias, and the need for appropriate human oversight [16]. Effective AI integration in public health therefore requires alignment between infrastructure, data governance, workforce capacity, organizational processes, regulation, and public health priorities.

The existing research in Saudi Arabia has increasingly examined perceptions of AI in healthcare among different groups, including medical science students and the general public. For example, previous studies have explored medical students’ attitudes and perceptions of AI in healthcare [17,18], while other research has assessed the Saudi public’s perceptions and opinions on this issue [19,20]. These studies indicate a growing awareness of AI and generally positive views regarding its potential to support healthcare delivery. However, less empirical attention has been given to health system readiness for AI integration specifically within public health. This distinction is important because clinical AI often focuses on diagnosis, treatment support, or patient-level decision-making, whereas public health AI requires broader system coordination, population-level data use, cross-sector governance, and policy alignment. Therefore, understanding AI readiness in public health requires attention to the perspectives of stakeholders directly involved in healthcare delivery, health informatics, and policymaking.

Qualitative inquiry is well suited for exploring AI readiness because it captures the contextual, organizational, technical, ethical, and policy-related factors that shape implementation. Stakeholder perspectives can provide valuable insight into how AI is understood, which opportunities are prioritized, what risks are perceived, and which barriers may affect sustainable integration. This is particularly important in Saudi Arabia, where national strategies strongly support digital transformation, yet the practical conditions required for AI integration in public health remain insufficiently understood. Therefore, this study explored stakeholder perceptions of the conditions relevant to health system readiness for AI integration in public health in Saudi Arabia, with attention to infrastructure and interoperability, workforce capacity, data governance and ethics, regulatory considerations, organizational capacity, and perceived strategic opportunities. Accordingly, the study addressed the following research question: How do key stakeholders perceive the conditions that facilitate or constrain health system readiness for integrating AI into public health in Saudi Arabia, and what implementation priorities and strategic opportunities do they identify? By exploring these readiness conditions from multiple stakeholder perspectives, this study contributes to AI implementation science by identifying perceived system-level conditions that may influence the translation of AI from strategic ambition into public health practice. It also extends health system readiness research within the Saudi context and provides evidence that may inform future policy development, workforce planning, governance, and implementation strategies for responsible AI integration.

## 2. Materials and Methods

### 2.1. Study Design

This study used a qualitative descriptive design to explore stakeholder perceptions of conditions relevant to health system readiness for artificial intelligence (AI) integration in public health in Saudi Arabia. The study was not designed to objectively measure, score, or verify national readiness using organizational, technical, workforce, financial, regulatory, or infrastructure indicators. Rather, it explored stakeholders’ perceptions of existing conditions, implementation gaps, and priorities relevant to AI integration. A qualitative approach was considered appropriate as the study aimed to generate an in-depth understanding of stakeholder perspectives, perceived barriers, implementation conditions, and strategic opportunities related to AI readiness [21]. Semi-structured interviews were used to allow participants to describe their experiences and views in depth while enabling the researcher to explore emerging issues relevant to infrastructure, workforce capacity, data governance, ethics, and policy. The reporting in this study was assessed against the 32-item Consolidated Criteria for Reporting Qualitative Research (COREQ) checklist to support transparent reporting of the research team, study design, data collection, analysis, and findings [22]. The completed COREQ checklist is provided in Supplementary File S1.

### 2.2. Study Setting

The study was conducted in Saudi Arabia, where the health system is undergoing major digital transformation as part of Vision 2030 and the Health Sector Transformation Program. The setting was considered appropriate because Saudi Arabia has invested in digital health infrastructure, e-health services, virtual care, national health information systems, and data-driven innovation. These developments provide an important context for exploring stakeholder perceptions of conditions relevant to health system readiness for integrating AI into public health functions such as surveillance, prevention, population health monitoring, and decision support.

Participants were drawn from public-sector healthcare organizations across multiple regions of Saudi Arabia, including Riyadh, Makkah, the Eastern Province, Asir, and Tabuk. The organizational settings included tertiary hospitals, general hospitals, and primary healthcare facilities, providing perspectives from different levels of healthcare delivery and organizational contexts. This geographical and organizational variation supported the maximum-variation sampling strategy and enabled the study to capture a range of operational environments relevant to AI readiness.

### 2.3. Participants and Recruitment

A maximum-variation purposive sampling strategy was used to recruit participants with diverse professional perspectives relevant to AI readiness in the Saudi health system. Variation was sought across stakeholder groups, professional roles, and levels of professional experience to capture operational, technical, and policy perspectives. The study included 32 participants representing three stakeholder groups: healthcare professionals, health informatics experts, and policymakers or individuals involved in healthcare planning and decision-making. Participants held professional roles spanning healthcare and public health practice, health informatics, and healthcare policy and planning, including clinical and public health practitioners, health informatics specialists, consultants and managers, and professionals involved in healthcare policy, planning, and decision-making. All participants had direct professional experience relevant to public health, including public health practice, public health policy and planning, disease surveillance, epidemiology, health promotion, and population health. Public health expertise therefore extended across all three stakeholder groups and was not limited to participants categorized as healthcare professionals. Participants also had professional experience relevant to health system or AI implementation, including healthcare delivery, digital health and health informatics, organizational implementation, healthcare leadership, policy, planning, and decision-making. This combination of public health and implementation-related expertise provided a relevant basis for exploring operational, technical, managerial, and policy perspectives on conditions related to AI readiness.

Participants varied in their direct experience with AI: some had professional experience involving AI-related initiatives, digital health implementation, health data systems, informatics, or technology-enabled decision support, whereas others contributed perspectives based primarily on public health practice, healthcare delivery, policy, planning, or organizational decision-making. Participants were eligible if they were currently working in Saudi Arabia, were able to provide informed consent, had direct professional experience relevant to public health, and had professional experience relevant to at least one aspect of health system or AI implementation. Relevant public health experience included public health practice, public health policy or planning, disease surveillance, epidemiology, health promotion, or population health. Relevant implementation-related experience included healthcare delivery, digital health or health informatics, healthcare leadership, policy, planning, or decision-making. Individuals who did not meet both the public health and implementation-related experience requirements were excluded.

Potential participants were initially identified through professional networks and academic and healthcare contacts. Recruitment was subsequently supplemented by snowball sampling, whereby participants recommended other potentially eligible individuals with relevant expertise. Potential participants were contacted by WhatsApp, version 2.26.34.74 (WhatsApp LLC, Menlo Park, CA, USA, 2026), or email. Before participation, participants were informed of the researcher’s identity, university affiliation, academic role, and the purpose of the study. Eligible individuals received information about the study purpose, voluntary participation, and interview format and were given the opportunity to ask questions before participation. Written or verbal informed consent was obtained before each interview, depending on the interview mode and participant preference. A total of 40 individuals were approached for participation; three declined and five did not respond, resulting in 32 completed interviews. Reasons for declining participation were not recorded.

During the initial data-collection phase, recruitment and preliminary analysis proceeded iteratively. The author reviewed interview content, developing codes, and emerging patterns throughout recruitment to assess whether the dataset provided sufficient depth, breadth, and variation to address the research question. Toward the completion of the initial 27 interviews, later interviews largely reinforced previously identified patterns rather than substantially extending the developing analysis. Because the study used reflexive thematic analysis, this judgment was treated as an assessment of analytic sufficiency rather than as evidence that all possible meanings or themes had been exhausted. Analytic sufficiency was assessed across the overall dataset and not separately within each stakeholder group; therefore, no subgroup-specific claim of analytic sufficiency is made. Five additional interviews were subsequently conducted during manuscript revision to broaden the range of stakeholder perspectives; these interviews were incorporated into the expanded dataset, and the full dataset was reconsidered during analysis. The additional interviews contributed further variation and supporting evidence but did not substantially alter the higher-order thematic structure.

### 2.4. Data Collection

Data were collected through semi-structured interviews, allowing a consistent approach across participants while retaining flexibility to explore individual experiences, interpretations, and priorities in depth. The interview guide was developed in line with the study aim, the relevant literature on AI readiness and digital health implementation, and key dimensions of AI implementation readiness [8,11,23]. Rather than being based on a single formal framework, the guide drew on commonly identified readiness dimensions, including digital infrastructure and interoperability, workforce knowledge and skills, data governance, privacy and ethics, regulatory considerations, organizational capacity, and implementation opportunities. These dimensions were used as broad areas of inquiry to structure the interviews rather than as predetermined analytic themes. The open-ended format allowed participants to introduce issues, relationships, and implementation concerns beyond those anticipated in the interview guide.

Before formal data collection, the interview guide was reviewed by three experts—two health informatics academics and one public health researcher—to assess the relevance, clarity, and appropriateness of the questions. It was subsequently pilot-tested to evaluate question clarity, sequencing, and the overall interview process. Feedback from the expert review and pilot testing was used to refine the guide before formal data collection, and no substantive modifications were made to the interview guide during data collection. The same core interview questions were used across all three stakeholder groups to support consistency and comparability, while probing questions were tailored to participants’ professional roles and responses and were used to explore unanticipated issues raised during the interviews in greater depth. The final guide included open-ended questions addressing participants’ understanding of AI in public health, perceived AI readiness, digital infrastructure, interoperability, workforce capacity, training needs, data governance, privacy, ethical considerations, regulatory issues, organizational challenges, and future opportunities. All interviews were conducted by the author, who holds a PhD in Public Health and has training and experience in qualitative research and semi-structured interviewing. At the time of the study, the author was an Assistant Professor at Umm Al-Qura University and was male. Face-to-face interviews were conducted in private offices or meeting rooms at participants’ workplaces, while online interviews were conducted using Zoom, version 6.2.6 (Zoom Video Communications, Inc., San Jose, CA, USA). No non-participants were present during the interviews, and no repeat interviews were conducted. The interviewer was not involved in the participants’ supervision, employment, or performance evaluation and had no prior relationship with the participants before recruitment. The semi-structured interview guide is provided in Supplementary File S2.

Initial interviews were conducted between June and July 2026. Five additional interviews were conducted in August 2026 during manuscript revision to strengthen the breadth of stakeholder perspectives. These additional interviews followed the same eligibility criteria, interview guide, and data-collection procedures as the initial interviews and were incorporated into the full dataset for analysis. Interviews were conducted either face-to-face or online, depending on participant availability and preference, and were held in Arabic or English according to the participant’s preferred language. Of the 32 interviews, 30 were conducted in Arabic and two were conducted in English. Each interview lasted approximately 30–45 min and, with participants’ consent, was audio-recorded using Zoom, version 6.2.6 (Zoom Video Communications, Inc., San Jose, CA, USA), to support accurate transcription and analysis. Field notes were recorded after interviews, where appropriate, to document contextual observations, notable issues raised during the interview, and preliminary analytic reflections. These notes were reviewed during familiarization and coding to support interpretation of the interview data, identify issues requiring closer examination, and inform comparisons across participants; they were not analyzed as a separate dataset.

All recordings were transcribed verbatim by the author, with Arabic interviews transcribed in Arabic and English interviews transcribed in English. Arabic transcripts were coded and analyzed in the original Arabic language and were not translated into English before analysis. The two English-language transcripts were coded and analyzed in English. Coding was therefore undertaken in the original language of each interview. Selected Arabic quotations were translated into English for reporting by the author, who is fluent in both Arabic and English, and the translated quotations were independently reviewed by a professional English–Arabic translator. A back-translation procedure was also undertaken, whereby the English translations were translated back into Arabic and compared with the original Arabic quotations to identify and resolve any discrepancies in meaning. This process focused on ensuring semantic and conceptual equivalence and preserving participants’ intended meaning, tone, and context rather than relying on literal word-for-word translation. Reported quotations were drawn directly from the interview transcripts and were not condensed or paraphrased. Quotations from interviews conducted in English were reported from the original English transcripts without substantive alteration. For quotations translated from Arabic, minor linguistic adjustments were made only where necessary to produce grammatically clear and natural English while preserving the participants’ original meaning, emphasis, and tone. No substantive content was added, removed, or altered during translation or preparation of the quotations for reporting. Before analysis, all transcripts were anonymized by assigning participants unique identification codes and removing identifying information. Transcripts were returned to participants for review and correction before final analysis. The final themes and interpretations were not returned to participants for additional member checking. Digital recordings, transcripts, and study documents were stored securely and were accessible only to the author.

### 2.5. Data Analysis

Data were analyzed using reflexive thematic analysis, following Braun and Clarke’s six-phase approach [24]. The analysis combined deductive sensitivity to broad readiness dimensions with inductive engagement with participants’ accounts and was conducted in an iterative and reflexive manner to develop patterns of shared meaning across the dataset that captured participants’ perspectives on AI readiness in public health. In the first phase, the author became familiar with the data by reading and re-reading the transcripts and reviewing field notes. Initial observations were recorded to capture early impressions and recurring ideas. Analytic memos were maintained throughout coding and theme development to document emerging interpretations, relationships among codes, areas of uncertainty, and decisions regarding the refinement of themes and subthemes. An audit trail was also maintained to record key analytic decisions and changes to the coding and thematic structure across successive stages of analysis. In the second phase, initial codes were generated across the dataset. Coding and data management were conducted manually using Microsoft Word, version 16.112.3, and Excel, version 16.112.3 (Microsoft Corporation, Redmond, WA, USA); no dedicated qualitative data-analysis software was used. Coding followed a hybrid deductive–inductive approach. The broad readiness dimensions that informed the study aim and interview guide served as sensitizing domains during analysis, while coding within and across these domains was developed from participants’ accounts. The interview guide domains were therefore not automatically treated as analytic themes. Codes were generated from participants’ descriptions, experiences, and concerns, and new codes were created when issues extended beyond the specific topics anticipated in the interview guide. This approach enabled issues not anticipated in the interview guide to be incorporated into the analysis while maintaining sensitivity to dimensions relevant to the research question. In the third phase, related codes were grouped to identify candidate subthemes based on shared patterns of meaning. Codes were compared both within and across the broader readiness domains to identify relationships, similarities, and differences in participants’ accounts. For example, codes concerning regional differences, smaller facilities, connectivity, and variation in technical capacity contributed to the subtheme “Uneven Digital Maturity Across Settings”; concerns about professional autonomy, changing responsibilities, and fear of replacement contributed to “Professional Acceptance and Role Clarity”; and concerns regarding representation, data quality, and unequal model performance contributed to “Algorithmic Bias and Data Representativeness.” In the fourth phase, candidate themes and subthemes were revisited in relation to the coded extracts and the full dataset, with attention to coherence, variation, divergent accounts, and cases that challenged dominant patterns, as well as the interpretive meaning generated through the analysis. In the fifth phase, themes and subthemes were refined, named, and defined. This process involved clarifying the central meaning of each theme and subtheme and identifying how the patterns represented within them related to the overall research aim. In the final phase, the findings were written up with illustrative quotations selected to represent the identified patterns of meaning. Although the resulting higher-order thematic structure broadly corresponded with the readiness domains explored during data collection, the specific subthemes and interpretive findings were developed through iterative engagement with participants’ accounts rather than being predetermined by the interview guide. The final analytic structure comprised three higher-order themes concerning perceived readiness conditions—infrastructure readiness, workforce capacity, and data governance and ethics—and a fourth theme capturing perceived strategic opportunities for AI in public health.

As the sole researcher, the author was involved in participant recruitment, interviewing, coding, theme development, and interpretation. The author has an academic background in public health and experience in digital health research, which provided relevant contextual knowledge but could also influence the interpretation of participants’ accounts. Reflexivity was maintained throughout the study through analytic memoing, including explicit consideration of how the researcher’s professional background, assumptions, prior knowledge, and interactions with participants might shape data generation, coding, theme development, and interpretation. The audit trail was used to document analytic decisions and support transparency throughout this process.

### 2.6. Ethics Approval

This study was conducted in accordance with the Declaration of Helsinki and was approved by the Biomedical Research Ethics Committee at Umm Al-Qura University, Saudi Arabia (approval No. HAPO-02-K-012-2026-05-3493). Initial data collection began in June 2026, after ethical approval had been obtained. All participants were informed about the study purpose, voluntary participation, confidentiality, and their right to withdraw at any time without providing a reason. Informed consent was obtained before each interview, either in written or verbal form depending on the interview mode and participant preference, in accordance with the procedures approved by the ethics committee. Audio recordings were treated as potentially identifiable data and were stored securely with restricted access. Transcripts were de-identified by removing names and other direct identifiers and assigning unique participant codes. To reduce the risk of deductive identification, potentially identifying institutional or role-specific details were omitted or generalized where necessary in reporting. Audio recordings, transcripts, and study documents were stored on a password-protected and encrypted device accessible only to the author. The data will be retained for the period required under applicable institutional and ethical requirements and will be securely destroyed thereafter.

## 3. Results

### 3.1. Participant Characteristics

The study included 32 participants, representing healthcare professionals, health informatics experts, and policymakers or healthcare planning stakeholders. Health informatics experts formed the largest stakeholder group (*n* = 13), followed by healthcare professionals (*n* = 10) and policymakers or healthcare planning stakeholders (*n* = 9). The distribution across stakeholder groups reflected the maximum-variation purposive sampling strategy, which sought to capture operational, technical, managerial, and policy perspectives relevant to health system and AI implementation. The sampling strategy was intended to capture breadth of relevant expertise rather than achieve equal numerical representation across stakeholder groups. All participants had direct professional experience related to public health: their primary areas of experience included public health practice (*n* = 10), public health policy or planning (*n* = 11), disease surveillance (*n* = 2), epidemiology (*n* = 3), health promotion (*n* = 2), and population health (*n* = 4). Thus, public health experience was represented across the stakeholder groups rather than being limited to the healthcare professional subgroup. Most participants were male (*n* = 17), although the gender distribution was relatively balanced. The largest age groups were 30–39 years and 40–49 years (*n* = 11 each). Regarding educational qualifications, 14 participants held a bachelor’s degree, 10 held a master’s degree, and eight held a doctoral degree. Most participants had between 5 and 10 years of professional experience (*n* = 22). The demographic and professional characteristics of the participants are presented in Table 1.

### 3.2. Overview of Findings

The analysis was organized around four higher-order themes: infrastructure readiness, workforce capacity, data governance and ethics, and perceived strategic opportunities for AI in public health. The first three themes captured conditions that participants perceived as directly relevant to health system readiness, while the fourth captured anticipated public health applications and benefits associated with AI implementation. Within these higher-order themes, the analysis developed subthemes that captured more specific patterns of meaning in participants’ accounts regarding conditions perceived to facilitate or constrain AI integration into public health. Each theme comprised several subthemes representing specific readiness conditions, implementation considerations, or anticipated applications of AI in public health. Together, these themes reflect participants’ perceptions of progress associated with national digital health transformation as well as perceived gaps that may affect the sustainable implementation of AI in public health. Participants are identified by professional role, years of professional experience, stakeholder group, and a unique participant code: healthcare professionals (HP), health informatics experts (HIE), and policymakers or healthcare planning stakeholders (PM). Illustrative quotations were selected to demonstrate patterns identified across the dataset and, where relevant, variation in participants’ perspectives rather than to imply that individual findings were derived from a single participant. A detailed summary of the themes, subthemes, key interpretive findings, representative codes, illustrative quotations, and stakeholder groups is provided in Supplementary File S3.

Across the themes, participants did not portray the principal challenge as an absence of digital health development or national commitment to AI. Rather, their accounts highlighted a gap between national-level digital transformation and the operational conditions required to translate these foundations into AI-enabled public health practice. Existing digital platforms and health data were viewed as important foundations, but their value for AI was perceived as depending on interoperability across organizations, more consistent digital maturity across settings, workforce capability, and clearer governance and regulatory arrangements. These findings suggest that participants understood AI readiness less as the presence of individual technological components and more as the alignment of technical, organizational, workforce, and governance conditions across the health system.

#### 3.2.1. Theme 1: Infrastructure Readiness

##### Digital Foundations for AI Integration

Participants generally acknowledged that Saudi Arabia has made substantial progress in developing digital health infrastructure. National investments in electronic health records, health information exchange platforms, telemedicine services, and unified digital portals were viewed as important foundations for future AI integration. Participants linked this progress to the broader policy environment created by Vision 2030 and the Health Sector Transformation Program, which were perceived to have accelerated digital transformation across the health system. One mid-level policymaker with 9 years of professional experience described these developments as an important starting point for AI adoption: “We have made clear progress in building digital platforms over the past several years. The national health portals and electronic record systems are important foundations. AI needs these foundations to function.” PM01. However, this positive assessment was not uniform. Some participants distinguished national-level progress from readiness within individual healthcare organizations. A health informatics expert with 8 years of professional experience explained: “I think nationally we are moving very fast, especially with Vision 2030. But when you look at individual hospitals, the situation is different. Some are ready, and others still have a lot of work to do.” HIE09.

These accounts indicate that participants distinguished progress in national digital transformation from the operational readiness of individual healthcare organizations, suggesting that system-level advancement had not necessarily produced uniform implementation capacity across settings.

##### Interoperability and Data Integration

Despite these advances, participants consistently identified interoperability and data integration as major barriers to AI readiness. Many described a fragmented digital environment in which health data exist across multiple facilities, sectors, and administrative systems but are not always connected in ways that support real-time public health analysis. Interoperability was viewed as technically complex, especially when different organizations use different platforms, data standards, or legacy systems. A health informatics consultant with 10 years of professional experience explained: “The problem is not that data does not exist. It exists in many places. The problem is that the systems do not speak to each other properly. In public health, we need data from hospitals, primary care, laboratories, and pharmacies to come together.” HIE02. Participants further distinguished the availability of large volumes of health data from their suitability for AI applications. A health informatics expert with 8 years of professional experience explained: “Having a lot of data does not mean the data are ready for AI. Sometimes there are missing data, different formats, or different ways of recording the same information.” HIE09.

Together, these accounts suggest that participants viewed AI-ready infrastructure as requiring more than the existence of digital data. Data also needed to be sufficiently complete, standardized, interoperable, and usable across organizational boundaries for AI-supported public health analysis.

##### Uneven Digital Maturity Across Settings

Participants raised concerns about uneven digital maturity across healthcare organizations and geographic areas. Some participants suggested that larger and more resourced institutions may be better prepared for AI implementation, while other settings may require further investment in connectivity, technical capacity, system standardization, and digital workflows. Participants considered this variation important because they viewed system-wide readiness, rather than readiness within isolated institutions, as necessary for public health AI implementation. An experienced clinician with 12 years of professional experience highlighted the risk of unequal readiness: “If we build AI tools only for the most advanced settings and forget the regions or smaller facilities, we may increase the gap instead of closing it.” HP03. A healthcare professional with 10 years of professional experience similarly described variation in digital maturity across healthcare settings: “In some hospitals, the systems are advanced and everything is electronic, but in other places we still face delays, disconnected systems, or manual work. This difference will affect how easily AI can be introduced.” HP07.

Taken together, these accounts suggest that participants viewed infrastructure readiness not simply as the availability of digital systems, but as the capacity to connect those systems across organizations and geographic settings in ways that support population-level analysis. They also indicated that national progress in digital health had not necessarily translated into an equal level of operational readiness across healthcare settings.

#### 3.2.2. Theme 2: Workforce Capacity

##### AI Literacy and Understanding

Workforce capacity emerged as one of the most prominent concerns across participant groups. Participants emphasized that AI readiness depends not only on technical systems but also on whether healthcare professionals, public health workers, informatics experts, managers, and policymakers have the knowledge and confidence to use AI appropriately. Limited AI literacy was described as a key barrier to effective implementation. Participants noted that many professionals may be aware of AI as a concept but have limited understanding of how AI systems work, what their outputs mean, and how their limitations should be evaluated in practice. This gap was identified among frontline professionals as well as those involved in planning and management. A health informatics specialist with 8 years of professional experience stated: “Most professionals have not been trained to work with AI tools. They may hear about AI or use systems that include AI, but they may not understand what AI can and cannot do. This is an important gap.” HIE01.

A healthcare professional with 6 years of professional experience similarly distinguished awareness of AI from the practical ability to interpret and use its outputs: “Many healthcare professionals know about AI, but using it in practice is different. We need training to understand the results and know when we can rely on them and when we should question them.” HP08.

##### Role-Specific Training and Practical Competencies

Participants emphasized the need for structured and practical training programs and argued that AI training should go beyond general digital skills and should include interpretation of AI outputs, data quality, bias, ethical use, and the role of human judgment. Participants also noted that public health professionals need to understand how AI-generated insights can be applied to surveillance, prevention, population health planning, and resource allocation. A senior policymaker with 17 years of professional experience explained: “We need professionals who can look at an AI output and ask the right questions. Where did the data come from? How was the model trained? What might it miss? What should we do if the result is wrong?” PM02.

Some differences in emphasis were apparent across stakeholder perspectives. A policymaker with 9 years of professional experience contrasted strong strategic support with unresolved technical requirements: “From the policy side, there is strong support for AI and digital transformation. But from the technical side, we still need to solve issues like interoperability, data quality, and standardization.” PM05.

##### Specialized Expertise and Workforce Retention

Several participants also discussed the need for specialized expertise in health data science, machine learning, and AI implementation. While participants recognized that technical talent exists in Saudi Arabia, they suggested that the healthcare and public health sectors need clearer career pathways, training opportunities, and retention strategies to attract and sustain this expertise. A health informatics manager with 16 years of professional experience commented: “The talent exists, but healthcare has to compete with other sectors. We need to make health and public health attractive areas for data scientists and AI specialists.” HIE03.

##### Professional Acceptance and Role Clarity

Participants across stakeholder groups also raised concerns about professional acceptance and role clarity. Some participants perceived that healthcare professionals may be concerned that AI could replace aspects of their work or reduce professional autonomy. Additional accounts from healthcare professionals directly reflected these concerns. Participants emphasized that AI should be introduced as a supportive tool that enhances decision-making rather than replaces human expertise. A healthcare professional with 11 years of professional experience explained: “If AI is introduced as something that supports our work, I think staff will accept it more. But if people feel it is replacing their judgment or controlling their decisions, there will be resistance.” HP06.

Participants also linked acceptance to workflow compatibility. A policymaker with 13 years of professional experience explained: “Even if the AI system is very good, if it makes the doctor’s or healthcare professional’s work more difficult, they will not accept it. It has to fit into their daily work.” PM06.

#### 3.2.3. Theme 3: Data Governance and Ethics

##### Privacy, Data Use, and Public Trust

Participants identified data governance and ethics as essential but still developing components of AI readiness, raising concerns about privacy, confidentiality, data access, security, consent, algorithmic bias, accountability, and regulation. These concerns were particularly important because AI in public health may rely on large-scale population data and may influence decisions affecting communities and vulnerable groups.

Participants emphasized that public trust is central to the use of AI in public health, noting that individuals and communities need confidence that health data are being used responsibly, securely, and for legitimate public health purposes. Governance was therefore viewed not only as a technical or administrative requirement but also as a condition for public acceptance. A healthcare policy advisor with 10 years of professional experience explained: “People need to trust that their data is being used to protect them, not to expose them. If that trust is lost, it will be difficult to move forward. Governance is what makes AI acceptable to society.” PM03.

##### Regulatory Clarity and Accountability

Participants indicated that existing data protection and health information policies may need to be further developed to address the specific characteristics of AI systems. Issues such as explainability, model validation, accountability for AI-supported decisions, and the monitoring of AI systems after deployment were viewed as requiring clearer guidance. A health informatics expert with 10 years of professional experience described this concern: “We have general data regulations, but AI in healthcare raises new questions. Who is responsible if an AI system gives a wrong prediction that affects a public health decision? How do we monitor a model after it is deployed?” HIE04.

Accountability was also identified as a practical professional concern. A healthcare professional with 9 years of professional experience stated: “As healthcare professionals, we need to know who is responsible if the AI gives the wrong recommendation. We cannot depend on a system without clear accountability.” HP09. However, views concerning regulation were not entirely uniform. A healthcare professional with 16 years of professional experience cautioned that regulatory processes could become burdensome if they were unnecessarily complex: “Of course, we need regulation because health data are sensitive. But if every AI project has to go through a very complicated process, this may slow down innovation.” HP10.

##### Algorithmic Bias and Data Representativeness

Algorithmic bias and data representativeness were also important concerns. Participants suggested that AI models trained on incomplete, poor-quality, or unrepresentative data could produce inaccurate or inequitable outputs. This was viewed as both an ethical and public health issue because biased AI outputs could affect risk prediction, service planning, and resource allocation. A healthcare professional with 10 years of professional experience stated: “If the data are biased, the AI output will also be biased. If some groups or facilities are overrepresented, the model may not work equally well for everyone.” HP05.

##### Multidisciplinary Governance and Oversight

Participants called for stronger AI governance frameworks that address data stewardship, ethical review, transparency, accountability, regulatory oversight, and public communication. They also emphasized the importance of involving multiple stakeholders, including public health professionals, health informatics experts, legal specialists, ethicists, policymakers, and community representatives. As a health informatics expert with 7 years of professional experience stated, “AI governance requires collaboration among policymakers, healthcare professionals, technical experts, legal specialists, and ethicists.” HIE06.

Governance was consequently described not as a separate administrative requirement, but as a condition linking technical implementation with accountability, public trust, and the legitimacy of using population-level data for AI-supported public health decisions. Participants’ accounts therefore positioned governance as a connecting mechanism between technical implementation, professional accountability, ethical safeguards, and public trust.

#### 3.2.4. Theme 4: Perceived Strategic Opportunities for AI in Public Health

This theme captured participants’ anticipated applications and potential benefits of AI rather than a separate dimension of current health system readiness. Despite the challenges identified, participants expressed optimism about the perceived strategic opportunities that AI could offer public health in Saudi Arabia. AI was viewed as a tool that could support a shift from reactive public health responses toward more proactive, preventive, and data-driven approaches. However, participants did not view these anticipated benefits as independent of readiness. Rather, their realization was perceived to depend on the availability of interoperable data, workforce capability, appropriate governance, regulatory clarity, and organizational capacity for implementation.

Although optimism predominated, participants did not uniformly favor rapid implementation. A healthcare professional with 16 years of professional experience expressed a more cautious perspective: “I am positive about AI, but I do not think we should rush. If the data are not ready and the rules are not clear, we may implement something that creates more problems.” HP10.

##### Preventive Care and Chronic Disease Management

Preventive care was one of the most frequently discussed opportunities. Participants suggested that AI could help identify individuals and communities at higher risk of disease, support early intervention, and improve the targeting of health promotion programs. Participants specifically referred to chronic diseases, including diabetes and hypertension, as important areas where AI could support earlier risk identification, targeted preventive interventions, and population-level monitoring. This was seen as particularly relevant in the context of chronic disease prevention and population health management. A healthcare professional with 10 years of professional experience emphasized the potential connection between early risk identification and prioritization of preventive care: “AI could be very useful in identifying high-risk patients earlier and helping us focus on people who need more attention, especially for chronic diseases and preventive care.” HP07.

##### Population Health Monitoring and Disease Surveillance

Population health monitoring and disease surveillance were also identified as important areas for AI application. Participants described the potential of AI to integrate multiple data sources, such as electronic health records, laboratory results, pharmacy data, surveillance systems, and environmental indicators, to provide early warning signals and support timely public health action. Participants also referred to the COVID-19 pandemic as an example of the importance of rapidly identifying and monitoring positive cases, highlighting the value of timely and integrated health data for disease surveillance and outbreak response. The pandemic was therefore viewed as an important example of why real-time, integrated data are essential for public health preparedness. A health informatics expert with 6 years of professional experience reflected: “The pandemic showed us the importance of seeing patterns across the system in real time. AI, if properly integrated, can help detect signals that may be missed in large volumes of daily data.” HIE05.

##### Decision Support and Resource Allocation

Participants also highlighted decision support as a key opportunity. AI was seen as potentially useful for public health planning, resource allocation, emergency preparedness, workforce planning, vaccination programs, screening initiatives, and evaluation of public health interventions. Participants noted that AI could support policymakers by providing timely insights and helping identify where resources may be most needed. As a health informatics expert with 9 years of professional experience stated, “AI can help decision-makers identify priority areas, allocate resources more efficiently, and respond more quickly to emerging public health needs.” HIE07.

##### Readiness Conditions for Realizing AI Benefits

However, participants were consistent in emphasizing that these opportunities require a strategic and phased approach. AI was not viewed as a standalone solution, but as a tool that needs to be integrated into broader health system transformation. Participants emphasized the need to align technical infrastructure, workforce capacity, governance, regulation, and public health priorities. These accounts indicate that the perceived value of AI was closely linked to implementation readiness: anticipated benefits were viewed as achievable only when the necessary technical, human, organizational, and governance conditions were in place. A mid-level policymaker with 10 years of professional experience summarized this point: “We have the ambition, the national strategy, and the beginning of the infrastructure. What we need now is alignment between the technology, the people, the governance, and the ethics. That is how AI can make a real difference in public health.” PM04.

A healthcare planning stakeholder with 8 years of professional experience similarly emphasized the interdependence of readiness conditions: “For me, you cannot look at these things separately. You may have good technology, but if the staff are not trained or the data are not connected or nobody knows who is responsible, the AI will not work properly.” PM07.

## 4. Discussion

### 4.1. Summary of the Main Findings

This study explored stakeholder perceptions of conditions relevant to health system readiness for integrating artificial intelligence into public health in Saudi Arabia. Four major themes were identified: infrastructure readiness, workforce capacity, data governance and ethics, and perceived strategic opportunities. Participants described national strategic ambition, investment in digital health, and ongoing transformation initiatives as favorable contextual conditions for AI integration, while at the same time perceiving important implementation challenges related to interoperability, workforce competencies, regulatory clarity, data governance, and organizational coordination. Overall, participants perceived a combination of strong national ambition for AI and uneven operational conditions across healthcare settings. Their accounts suggest that readiness is understood as a multidimensional implementation process requiring alignment between technology, workforce capacity, governance, organizational processes, and public health priorities. These findings represent stakeholder interpretations and should not be interpreted as an objective or comprehensive assessment of national health system readiness.

A central interpretation arising from these accounts is the tension between nationally coordinated digital transformation and uneven implementation conditions at the organizational level. While participants described strong national policy direction and rapid digital health development, they also indicated that these system-level developments did not necessarily translate into consistent operational capacity across healthcare organizations and geographic settings. In the Saudi context, the implementation challenge may therefore lie not only in establishing national strategies and digital infrastructure, but also in translating these system-level ambitions into interoperable data practices, workforce competencies, governance arrangements, and workable organizational processes across diverse settings. This distinction is important because centralized national coordination may facilitate rapid strategic and technological development, while effective implementation ultimately depends on the capacity of individual organizations to incorporate these changes into routine practice.

This study extends previous Saudi research on AI in healthcare in several important ways. Earlier studies have largely examined individual-level awareness, attitudes, perceptions, or acceptance of AI among students, healthcare-related groups, and the general public [17,18,20]. In contrast, the present study adopts a systems-level perspective by examining conditions perceived by stakeholders as relevant to AI implementation across infrastructure, workforce capacity, data governance and ethics, and perceived strategic opportunities. The inclusion of healthcare professionals, health informatics experts, and policymakers further enables a consideration of operational, technical, and policy-level perspectives within the same study. Rather than focusing primarily on whether stakeholders view AI positively or negatively, the findings identify organizational and implementation conditions perceived by stakeholders as important for safe, equitable, and sustainable AI integration into public health practice. The study therefore contributes a context-specific interpretation of how national strategic ambition may interact with uneven organizational implementation capacity within the Saudi health system.

#### 4.1.1. Infrastructure Readiness

Participants recognized progress in electronic health records, digital platforms, telemedicine, and health information systems, which provide an important foundation for AI integration. However, they consistently identified fragmented systems, limited interoperability, and difficulties integrating data from hospitals, primary care facilities, laboratories, and other sources. These findings align with previous studies showing that AI implementation in public health remains uneven because of limited infrastructure and insufficient technical understanding, while the availability of technical support and maintenance services is an important factor influencing successful adoption [2,11,25]. These findings indicate that the availability of digital data alone is insufficient: AI systems require standardized, accurate, timely, and connected data to support reliable public health surveillance and decision-making [26]. Uneven digital maturity between institutions and regions may also limit system-wide implementation and create disparities in access to AI-supported services. Participants’ accounts therefore suggest that strengthening interoperability standards and data-sharing mechanisms may represent an important implementation priority. The importance of these system foundations is also evident in other areas of healthcare. For example, a policy analysis of pediatric medication safety emphasized standardized protocols and EHR-supported care coordination, while highlighting the need for adequate IT infrastructure, staff training, data security, and equitable access across healthcare facilities [27].

#### 4.1.2. Workforce Capacity

Limited AI literacy and practical training emerged as major barriers. Participants reported that many healthcare and public health professionals were familiar with AI as a concept but lacked sufficient understanding of how AI systems work, how outputs should be interpreted, and how potential errors or bias should be assessed. This finding is consistent with previous research indicating that countries such as Saudi Arabia face context-specific challenges in public healthcare related to AI literacy, understanding, and the effective use of AI technologies [11]. In addition, research involving health information professionals found that many had limited or no practical experience with AI, while those with direct AI experience performed better on foundational knowledge questions, suggesting that experiential learning can strengthen AI literacy [23]. These findings suggest that workforce readiness involves more than increasing general awareness of AI. Participants emphasized the capacity to interpret AI outputs critically, recognize uncertainty and bias, preserve professional judgment, and incorporate AI appropriately into existing workflows. Professional acceptance also appeared to depend not only on knowledge of AI but on whether implementation was perceived to support rather than constrain professional roles and decision-making. Workforce preparation may therefore need to be considered alongside workflow design, role clarity, and organizational implementation rather than as an isolated training issue.

The findings highlight the need for role-specific education rather than general awareness programs. Public health professionals require skills in interpreting AI outputs, evaluating data quality, understanding bias and uncertainty, and applying findings alongside professional judgment. Policymakers and managers also require competencies in procurement, governance, evaluation, and risk management. A comparable emphasis on specialized professional capability, appropriate monitoring, and decision support has been reported in other complex healthcare contexts. Research involving specialists in pediatric pharmacotherapy has highlighted the need for careful treatment optimization, enhanced monitoring, and clinical decision support to manage complex treatment-related risks [28]. Although this evidence relates to a different clinical context, it similarly illustrates the broader importance of specialized professional capability when technology-supported decisions involve complex risks. Practical exposure, case-based learning, and hands-on training could therefore be incorporated into professional development programs to support the effective and responsible use of AI. Participants also linked workforce readiness to the availability and retention of specialized expertise in health data science, machine learning, and AI implementation, suggesting that workforce planning may need to extend beyond training the existing workforce to include career pathways and retention strategies that enable healthcare and public health organizations to attract and sustain specialized technical expertise.

#### 4.1.3. Data Governance and Ethics

Data governance and ethics were viewed as essential conditions for AI readiness. Participants raised concerns regarding privacy, confidentiality, cybersecurity, consent, algorithmic bias, explainability, accountability, and regulation. These concerns are particularly important in public health because AI may rely on large population datasets and influence decisions affecting entire communities. This finding is consistent with previous research showing that AI implementation in public health remains uneven because of data limitations and ethical and privacy concerns [2]. Other studies have identified insufficient patient data privacy measures as a major barrier and have reported substantial operational, tactical, and strategic challenges related to data protection and compliance with regulatory frameworks [25].

Participants’ accounts also revealed a tension between the need for stronger governance and the concern that overly complex regulatory processes could impede implementation. This suggests that the governance challenge is not simply to increase regulation, but to develop proportionate arrangements that provide accountability, privacy protection, transparency, and oversight without creating unnecessary barriers to responsible innovation. In this sense, governance was perceived not only as a safeguard but also as an enabling condition for organizational trust and implementation.

Algorithmic bias was also identified as a major risk. Models developed using incomplete or unrepresentative data may produce unequal outcomes across populations or regions. This concern is supported by recent literature highlighting the potential for AI algorithms to reproduce or amplify existing biases and healthcare disparities [29]. Governance frameworks may therefore benefit from including regular assessments of model performance, fairness, transparency, and public health impact, while multidisciplinary oversight and public engagement may further strengthen accountability and trust.

#### 4.1.4. Perceived Strategic Opportunities

These opportunities should be interpreted as participants’ anticipated applications and potential benefits of AI rather than as evidence of current health system readiness. Despite these challenges, participants were optimistic about the potential of AI to improve public health. Key opportunities included preventive care, chronic disease risk prediction, disease surveillance, early outbreak detection, population health monitoring, resource allocation, emergency preparedness, and decision support. These findings are consistent with previous research showing that AI can improve the efficiency, accuracy, and scalability of disease surveillance by automating data collection, strengthening outbreak prediction, and enabling real-time health monitoring [2,30]. AI-driven predictive models have also been used to identify emerging health threats, optimize resource allocation, and strengthen public health response mechanisms. Other applications reported in the literature include spatial modeling, risk prediction, misinformation control, disease forecasting, pandemic and epidemic modeling, surveillance, and health diagnosis [2,30].

Although participants’ accounts suggest that AI may help support a shift from reactive healthcare toward more preventive and data-driven public health practice in Saudi Arabia, predictive information has limited value unless healthcare organizations have the capacity to act on it. Participants’ accounts therefore suggest that future AI initiatives would need to be linked to clear interventions, referral pathways, workforce capacity, and measurable public health outcomes. Participants emphasized that implementation should be phased and focused on clearly defined public health problems and suggested that priority should be given to applications supported by high-quality data, clear public benefit, manageable ethical risks, and strong human oversight. Evaluation may therefore need to extend beyond technical accuracy to include equity, acceptability, workflow impact, and sustainability.

The contribution of this study does not lie in identifying interoperability, workforce capability, or governance as entirely new challenges, as these issues are well established in the international literature. Rather, the Saudi data provide context-specific empirical insight into how these conditions intersect within a rapidly transforming and centrally coordinated health system. In particular, participants’ accounts highlight a tension between strong national strategic ambition and variable organizational capacity to translate that ambition into practice. The findings also suggest that infrastructure, workforce capability, and governance should not be considered as independent readiness domains: limitations in one area may constrain progress in others. For example, interoperable data have limited value without professionals able to interpret AI outputs, while workforce capability alone cannot support responsible implementation without clear governance and accountability arrangements. The study therefore provides an implementation-oriented understanding of AI readiness as an interconnected set of organizational conditions rather than a checklist of isolated technical requirements. From a policy perspective, the findings suggest that national AI strategies may need to be accompanied by organization-level implementation mechanisms, including interoperable data practices, role-specific workforce development, proportionate governance, and phased implementation around clearly defined public health problems. This may be particularly relevant to health systems undergoing rapid, nationally coordinated digital transformation.

### 4.2. International Comparison and Contextual Implications

Several perceived readiness challenges identified by the participants in this study have also been reported in other high-income health systems. A qualitative study of information governance professionals in the United Kingdom similarly identified challenges related to variation in AI knowledge, data quality, fragmented organizational structures, governance and regulatory requirements, and implementation processes [31]. In the United States, a recent survey of major health systems found substantial variation in the adoption and perceived success of AI applications, with immature AI technologies, financial concerns, and regulatory uncertainty identified as major barriers to implementation [32]. These similarities indicate that concerns regarding workforce capability, governance, organizational coordination, and regulatory preparedness are also reported in other health systems and are not unique to the Saudi context.

However, the Saudi context is characterized by a rapid pace of nationally coordinated digital transformation and a strong strategic commitment to digital health and AI. Within this context, participants generally perceived these national developments as creating favorable conditions for AI adoption, while also identifying uneven operational conditions across healthcare settings. What may distinguish the Saudi context is therefore not the existence of interoperability, workforce, or governance challenges themselves, but the institutional setting in which they occur: strong centralized strategic direction and rapid national digital investment alongside variation in organizational implementation capacity. The key challenge may therefore be translating national ambition into coordinated implementation across digital infrastructure, workforce capacity, governance, regulation, and public health practice. This tension between national coordination and local implementation capacity provides an important context for interpreting participants’ accounts and distinguishes the Saudi implementation environment from some of the international settings discussed above.

The findings should also be interpreted within the contextual boundaries of the Saudi health system. Saudi Arabia is characterized by strong national policy direction, substantial investment in digital health, and a relatively centralized approach to health system transformation. These conditions may differ from those in more decentralized, resource-constrained, or fragmented health systems. Accordingly, findings related to national coordination, interoperability, governance, and implementation capacity may be most transferable to health systems undergoing similarly rapid digital transformation or pursuing nationally coordinated AI strategies. In contrast, health systems with limited digital infrastructure, different regulatory arrangements, greater organizational fragmentation, or substantially different workforce and financing structures may face additional or different readiness challenges. The findings should therefore be considered as contextually situated rather than assumed to apply uniformly across health systems.

### 4.3. Strengths and Limitations

This study provides an in-depth exploration of stakeholder perceptions of conditions relevant to health system readiness for AI integration in public health within the Saudi Arabian context, an area that remains underexplored. A key strength was the inclusion of healthcare professionals, health informatics experts, and policymakers or healthcare planning stakeholders, which enabled the study to capture diverse perspectives across operational, technical, managerial, and policy levels. The use of semi-structured interviews also allowed participants to discuss complex issues related to infrastructure, workforce capacity, governance, ethics, and implementation opportunities in detail.

Despite these strengths, several limitations should be considered. First, this qualitative study was based on a purposively selected sample of 32 participants and was designed to explore stakeholder perceptions rather than objectively measure national or organizational AI readiness. The findings should therefore not be interpreted as evidence of the actual readiness of the Saudi health system. Rather, their transferability to other settings should be considered in relation to similarities in organizational, professional, and health system contexts. Recruitment through professional and academic networks, supplemented by snowball sampling, may also have introduced selection bias by attracting participants with greater interest or experience in digital health, AI, or health system transformation and may have limited the range of perspectives represented in the sample.

Second, social-desirability bias may have influenced participants’ accounts, particularly when discussing national transformation initiatives, organizational performance, or institutional policies. Some participants may have been reluctant to express strongly critical views about their organizations or national initiatives. Although confidentiality was emphasized and potentially identifying institutional details were omitted or generalized, the possibility that participants moderated some of their responses cannot be excluded.

Third, although all participants had direct professional experience relevant to public health, public health expertise was distributed across different professional and stakeholder roles rather than represented by a large, dedicated subgroup of public health specialists. The stakeholder groups were also not equal in size, comprising 10 healthcare professionals, 13 health informatics experts, and nine policymakers or healthcare planning stakeholders. Consequently, the findings should not be interpreted as representing each stakeholder group equally or comprehensively. Analytic sufficiency was considered across the overall dataset rather than separately within each stakeholder group; therefore, subgroup-specific comprehensiveness is not claimed. In addition, the study focused on professional and policy stakeholders and did not include patients, members of the public, or community representatives. Their perspectives may differ, particularly regarding trust, privacy, consent, equity, acceptability, and the use of population health data for AI-supported public health activities. Future research should therefore incorporate patient and community perspectives alongside professional stakeholders.

Fourth, most interviews were conducted in Arabic and analyzed in Arabic, with selected quotations subsequently translated into English. Although translation involved independent bilingual review and back-translation to support semantic and conceptual equivalence, some linguistic or cultural nuance may have been altered during translation.

Fifth, as this was a single-researcher study, the author conducted the interviews, coding, theme development, and interpretation. The researcher’s academic background in public health and experience in digital health research may have influenced data generation and interpretation. Reflexive analytic memoing and an audit trail were used to document emerging interpretations, analytic decisions, and the potential influence of the researcher’s assumptions and prior knowledge; nevertheless, alternative interpretations of the data remain possible.

Finally, the study did not incorporate objective indicators of health system AI readiness, such as technical infrastructure assessments, interoperability measures, workforce competency assessments, organizational performance indicators, or implementation outcomes. The findings therefore represent stakeholder perceptions of readiness-related conditions rather than an objective or validated assessment of actual health system readiness. Future research could combine qualitative stakeholder perspectives with organizational and system-level readiness measures and include broader representation of frontline professionals, patients, and community stakeholders across different regional and organizational settings.

## 5. Conclusions

Stakeholders perceived substantial national strategic commitment and digital health development alongside important implementation challenges related to interoperability, workforce competencies, data governance, privacy, algorithmic bias, and regulatory clarity. Participants also identified potential opportunities for AI to support disease surveillance, preventive care, population health monitoring, resource allocation, and public health decision-making. These findings represent stakeholder perceptions of readiness conditions and should not be interpreted as an objective assessment of national health system readiness.

These stakeholder perspectives highlight four priorities that may warrant consideration in future policy and implementation. First, strengthening national interoperability and data-sharing standards may support the secure and reliable integration of data across healthcare settings. Second, role-specific AI literacy and training programs may help strengthen relevant competencies among public health professionals, healthcare workers, managers, and policymakers. Third, clearer governance and ethical review frameworks may help address issues such as privacy, accountability, transparency, algorithmic bias, model validation, and regulatory oversight. Fourth, participants’ accounts support consideration of phased, problem-focused AI implementation accompanied by human oversight and continuous evaluation of effectiveness, equity, acceptability, workflow impact, and sustainability. Aligning these priorities with national public health goals may support safer, more equitable, and more effective AI implementation within the Saudi health system. Because perspectives on public trust, privacy, consent, and acceptability in this study were reported by professional and policy stakeholders, future research should directly engage with patients, members of the public, and community representatives to examine these issues from their perspectives.

## Figures and Tables

**Table 1:** Demographic and professional characteristics of participants (*n* = 32).

Characteristic	Category	*n* (%)
**Stakeholder group**	Healthcare professionals	10 (31.3)
	Health informatics experts	13 (40.6)
	Policymakers/healthcare planning stakeholders	9 (28.1)
**Primary area of public health experience**	Public health practice	10 (31.3)
	Public health policy/planning	11 (34.4)
	Disease surveillance	2 (6.3)
	Epidemiology	3 (9.4)
	Health promotion	2 (6.3)
	Population health	4 (12.5)
**Gender**	Male	17 (53.1)
	Female	15 (46.9)
**Age group**	20–29 years	7 (21.9)
	30–39 years	11 (34.4)
	40–49 years	11 (34.4)
	≥50 years	3 (9.4)
**Highest qualification**	Bachelor’s degree	14 (43.8)
	Master’s degree	10 (31.3)
	Doctoral degree	8 (25.0)
**Professional experience**	<5 years	2 (6.3)
	5–10 years	22 (68.8)
	>10 years	8 (25.0)

## Data Availability

The data presented in this study are available on reasonable request from the corresponding author due to confidentiality restrictions and the sensitive nature of the participants’ professional views.

## Supplementary Materials

The following supporting information can be downloaded at: https://www.mdpi.com/article/10.3390/healthcare14183008/s1. Supplementary File S1: Completed COREQ checklist; Supplementary File S2: Interview Guide; Supplementary File S3: Summary of themes, subthemes, key interpretive findings, representative codes, illustrative quotations, and stakeholder groups.
