# Supplementary material for: Stakeholder Perspectives on Health System Readiness for Integrating Artificial Intelligence into Public Health in Saudi Arabia
Source: Healthcare (Basel). 2026 Sep 14;14(18):3008. doi: 10.3390/healthcare14183008 (PMC13606694; doi:10.3390/healthcare14183008)
Supplement: Supplementary file 1 [file healthcare-14-03008-s001.zip › Supplementary File S2.pdf]

Supplementary File S2

Interview Guide

Stakeholder Perspectives on Health System Readiness for Integrating Artificial Intelligence into Public Health in Saudi Arabia

Opening Statement

Thank you for agreeing to participate in this interview. The purpose of this study is to explore stakeholder views on the conditions relevant to integrating artificial intelligence (AI) into public health in Saudi Arabia. The interview will focus on your experiences, perspectives, concerns, and priorities related to this topic. This study was conducted in accordance with the Declaration of Helsinki and was approved by the Biomedical Research Ethics Committee at Umm Al-Qura University, Saudi Arabia (approval No. HAPO-02-K-012-2026-05-3493).

Your participation is voluntary. You may decline to answer any question or stop the interview at any time. With your permission, the interview will be audio-recorded to support accurate transcription and analysis. All information will be treated confidentially, and identifying information will not be used in reports or publications.

Do you have any questions before we begin?

The questions served as broad areas of inquiry, with probes used flexibly to clarify or expand responses.

Section 1: Participant Background

Can you briefly describe your current role and professional background?

How many years of professional experience do you have?

What type of organization or sector do you currently work in?

Section 2: General Views on AI in Public Health

What does artificial intelligence in public health mean to you?

How do you think AI may be relevant to public health in Saudi Arabia?

What factors do you think are important when considering whether a health system is prepared to use AI?

How would you describe the current situation in Saudi Arabia regarding the use or future use of AI in public health?

Section 3: Technology and Health Data

What are your views on the current digital and technical environment for supporting AI in public health?

What issues related to health data may influence the use of AI?

Section 4: Workforce and Organizations

How prepared do you think people working in healthcare and public health are for the use of AI?

What kinds of knowledge, skills, or support may be needed?

How might AI affect the way professionals work or make decisions?

What organizational factors may support or hinder the use of AI in public health?

Section 5: Governance, Ethics, and Policy

What governance or policy issues or ethical concerns should be considered when using AI in public health?

What concerns may arise regarding the use, protection, or sharing of health data?

What responsibilities or safeguards should be in place when AI is used to support public health decisions?

Section 6: Opportunities and Challenges

What potential benefits or opportunities do you see for AI in public health?

What challenges or risks could make AI difficult to implement or use effectively?

Are there particular areas of public health where you think AI may be especially useful? Why?

In your view, what would successful and responsible AI integration into public health look like in Saudi Arabia?

Closing Questions

Is there anything else you would like to add about AI integration into public health in Saudi Arabia?

Closing Statement

Thank you for your time and valuable insights. Your participation is greatly appreciated.
