# Supplementary material for: Stakeholder Perspectives on Health System Readiness for Integrating Artificial Intelligence into Public Health in Saudi Arabia
Source: Healthcare (Basel). 2026 Sep 14;14(18):3008. doi: 10.3390/healthcare14183008 (PMC13606694; doi:10.3390/healthcare14183008)
Supplement: Supplementary file 1 [file healthcare-14-03008-s001.zip › Supplementary File S3.pdf]

Supplementary File S3

Summary of themes, subthemes, key interpretive findings, representative codes, illustrative quotations, and stakeholder groups.

Theme	Subtheme	Key interpretive finding	Representative codes	Illustrative quotation	Stakeholder group
**1. Infrastructure Readiness**	**Digital Foundations for AI Integration**	Participants perceived national investment in EHRs, digital platforms, telemedicine, and health-information exchange as important foundations for AI, while distinguishing national digital progress from readiness at the organizational level.	National digital platforms; EHR development; telemedicine; digital transformation; national strategic support; national–organizational readiness gap	"We have made clear progress in building digital platforms over the past several years. The national health portals and electronic record systems are important foundations. AI needs these foundations to function." (PM01)	PM
	**Interoperability and Data Integration**	The existence of large volumes of digital health data was not considered sufficient for AI readiness; data needed to be connected, standardized, complete, and usable across organizational boundaries.	Fragmented systems; incompatible platforms; data integration; inconsistent standards; legacy systems; data quality; inconsistent data formats	"The problem is not that data does not exist. It exists in many places. The problem is that the systems do not speak to each other properly. In public health, we need data from hospitals, primary care, laboratories, and pharmacies to come together." (HIE02)	HIE
	**Uneven Digital Maturity Across Settings**	Participants perceived substantial variation in technical capacity and digital maturity across organizations and regions, raising concerns that AI implementation could reproduce or widen existing disparities.	Regional differences; smaller facilities; connectivity gaps; technical-capacity variation; manual workflows; unequal digital maturity	"In some hospitals, the systems are advanced and everything is electronic, but in other places we still face delays, disconnected systems, or manual work. This difference will affect how easily AI can be introduced." (HP07)	HP
**2. Workforce Capacity**	**AI Literacy and Understanding**	General awareness of AI did not necessarily translate into the practical knowledge needed to interpret AI outputs, understand limitations, or use AI appropriately in practice.	Limited AI literacy; awareness–practice gap; limited practical experience; interpretation of outputs; understanding AI limitations	"Many healthcare professionals know about AI, but using it in practice is different. We need training to understand the results and know when we can rely on them and when we should question them." (HP08)	HP
	**Role-Specific Training and Practical Competencies**	Participants emphasized practical, role-specific preparation rather than generic AI awareness, including competencies in interpreting outputs, evaluating data quality and bias, and applying AI appropriately to public health decisions.	Role-specific training; practical competencies; interpreting AI outputs; data-quality assessment; bias recognition; ethical use; professional judgment	"We need professionals who can look at an AI output and ask the right questions. Where did the data come from? How was the model trained? What might it miss? What should we do if the result is wrong?" (PM02)	PM
	**Specialized Expertise and Workforce Retention**	Sustainable AI implementation was perceived to require specialized expertise in health data science, machine learning, and implementation, together with career pathways and retention strategies.	Data-science expertise; machine-learning skills; specialized AI workforce; career pathways; competition for talent; workforce retention	"The talent exists, but healthcare has to compete with other sectors. We need to make health and public health attractive areas for data scientists and AI specialists." (HIE03)	HIE

	**Professional Acceptance and Role Clarity**	Acceptance was perceived to depend on presenting AI as a tool that supports rather than replaces professional expertise and ensuring that AI fits existing roles and workflows.	Fear of replacement; professional autonomy; role clarity; supportive AI; workflow compatibility; professional acceptance	"Even if the AI system is very good, if it makes the doctor's or healthcare professional's work more difficult, they will not accept it. It has to fit into their daily work." (PM06)	PM
**3. Data Governance and Ethics**	**Privacy, Data Use, and Public Trust**	Responsible and legitimate use of population health data was perceived as central to trust and acceptance, with privacy, confidentiality, security, and consent forming important governance concerns.	Privacy; confidentiality; data security; consent; responsible data use; legitimacy; public trust	"People need to trust that their data is being used to protect them, not to expose them. If that trust is lost, it will be difficult to move forward. Governance is what makes AI acceptable to society." (PM03)	PM
	**Regulatory Clarity and Accountability**	Participants perceived a need for clearer accountability, validation, explainability, and post-deployment monitoring requirements while also cautioning against unnecessarily burdensome regulation.	Regulatory clarity; accountability; explainability; model validation; post-deployment monitoring; responsibility for errors; regulatory burden	"We have general data regulations, but AI in healthcare raises new questions. Who is responsible if an AI system gives a wrong prediction that affects a public health decision? How do we monitor a model after it is deployed?" (HIE04)	HIE
	**Algorithmic Bias and Data Representativeness**	Poor-quality, incomplete, or unrepresentative data were perceived as potential sources of biased AI outputs and inequitable public health decisions.	Data representativeness; incomplete data; biased data; unequal model performance; fairness; equity risks	"If the data are biased, the AI output will also be biased. If some groups or facilities are overrepresented, the model may not work equally well for everyone." (HP05)	HP
	**Multidisciplinary Governance and Oversight**	Effective governance was viewed as requiring collaboration among technical, clinical, policy, legal, ethical, and community stakeholders rather than being treated as a purely administrative function.	Multidisciplinary oversight; data stewardship; ethical review; regulatory oversight; stakeholder collaboration; public communication	"AI governance requires collaboration among policymakers, healthcare professionals, technical experts, legal specialists, and ethicists." (HIE06)	HIE
**4. Perceived Strategic Opportunities for AI in Public Health**	**Preventive Care and Chronic Disease Management**	Participants anticipated that AI could support earlier identification of high-risk individuals and more targeted prevention, particularly for chronic diseases such as diabetes and hypertension.	Risk prediction; high-risk identification; early intervention; chronic disease prevention; targeted health promotion; population monitoring	"AI could be very useful in identifying high-risk patients earlier and helping us focus on people who need more attention, especially for chronic diseases and preventive care." (HP07)	HP
	**Population Health Monitoring and Disease Surveillance**	AI was perceived as potentially supporting real-time analysis of multiple data sources, early warning signals, disease surveillance, and outbreak response.	Real-time monitoring; multi-source data integration; early warning; outbreak detection; disease surveillance; pandemic preparedness	"The pandemic showed us the importance of seeing patterns across the system in real time. AI, if properly integrated, can help detect signals that may be missed in large volumes of daily data." (HIE05)	HIE
	**Decision Support and Resource Allocation**	Participants perceived AI as potentially useful for prioritizing public health needs and supporting resource allocation, preparedness, workforce planning, vaccination,	Decision support; priority identification; resource allocation; emergency preparedness; workforce planning; vaccination planning; screening	"AI can help decision-makers identify priority areas, allocate resources more efficiently, and respond more quickly to emerging public health needs." (HIE07)	HIE

		screening, and intervention planning.			
	**Readiness Conditions for Realizing AI Benefits**	Anticipated AI benefits were viewed as conditional on alignment among infrastructure, workforce capability, governance, regulation, and organizational implementation; participants favored strategic and phased implementation rather than AI adoption as a standalone solution.	Phased implementation; readiness alignment; infrastructure–workforce interdependence; governance alignment; regulatory clarity; organizational capacity	"For me, you cannot look at these things separately. You may have good technology, but if the staff are not trained or the data are not connected or nobody knows who is responsible, the AI will not work properly." (PM07)	PM

Note: HP = healthcare professional; HIE = health informatics expert; PM = policymaker or healthcare planning stakeholder. The stakeholder group column refers to the participant providing the illustrative quotation, rather than implying that the subtheme was identified exclusively within that stakeholder group.
